# Arabic translation, cultural adaptation, and validation of the BDDQ-AS for rhinoplasty patients

**DOI:** 10.1186/s40463-022-00613-6

**Published:** 2023-02-09

**Authors:** Ahmed S. Abdelhamid, Saad Elzayat, Mohamed A. Amer, Hossam S. Elsherif, Garyfalia Lekakis, Sam P. Most

**Affiliations:** 1 Department of Otorhinolaryngology-Head and Neck Surgery, Faculty of Medicine, Kafrelsheikh University, El-Geish Street, Kafrelsheikh, 33155 Egypt; 2 Department of Otorhinolaryngology, Faculty of Medicine, Tanta University, El-Gesih St., Tanta, Egypt; 3 Department of Otorhinolaryngology, Head and Neck Surgery, Moliere Longchamp Hospital, Rue Marconi 142, 1190 Brussels, Belgium; 4 Division of Facial Plastic and Reconstructive Surgery, Department of Otolaryngology-Head and Neck Surgery, Stanford University School of Medicine, 801 Welch Road Palo Alto, Stanford, CA 94304 USA

**Keywords:** Rhinoplasty, Body dysmorphic disorder, Screening, Arabic, Questionnaire, BDD

## Abstract

**Background:**

Body Dysmorphic Disorder (BDD) is a significant aspect that compromises patient satisfaction after rhinoplasty. BDDQ-AS (Body Dysmorphic Disorder Questionnaire-Aesthetic Surgery) is a validated, simple, reliable patient-reported outcome measure. It is a screening tool to detect body dysmorphic disorder in rhinoplasty patients. This study aimed to translate, culturally adapt, and validate BDDQ-AS to Arabic as a novel tool for screening and detecting BDD in Arabic rhinoplasty individuals.

**Methods:**

BDDQ-AS was translated from English to Arabic following the international consensus guidelines. We tested the translation on ten Arabic-speaking rhinoplasty patients to ensure that the final version was understandable and acceptable. The proposed Arabic version was then completed by 112 patients whose average age was 28.79 ± 9.32 years. The screening is assumed positive if the patients expressed bother and preoccupation about their appearance (questions 1 and 2 “yes”), as well as a moderately disrupted everyday life (question 7 "yes" or questions 3, 4, 5, or 6 are equal to or greater than “3′′). The internal consistency, test–retest reliability, and item-response theory (IRT) were used to evaluate psychometric validations.

**Results:**

The Arabic BDDQ had a high level of internal consistency, as measured by Cronbach's alpha 0.995. The A-BDDQ-AS was deemed reliable with an Intraclass Correlation Coefficient (ICC) of 0.989. A-BDDQ had good discrimination scores (above 2.0) with adequate difficulty parameters. The overall scale content validity average was 0.83, affirming that all items were relevant, clear, and straightforward.

**Conclusion:**

The Arabic version of the BDDQ-AS is reliable, culturally adapted, and psychometrically validated to be readily used and incorporated into clinical practice. It is a beneficial tool that can guide the screening of Arabic rhinoplasty patients suffering from body dysmorphic disorder and be utilized in further studies to optimize patient outcomes.

**Graphical Abstract:**

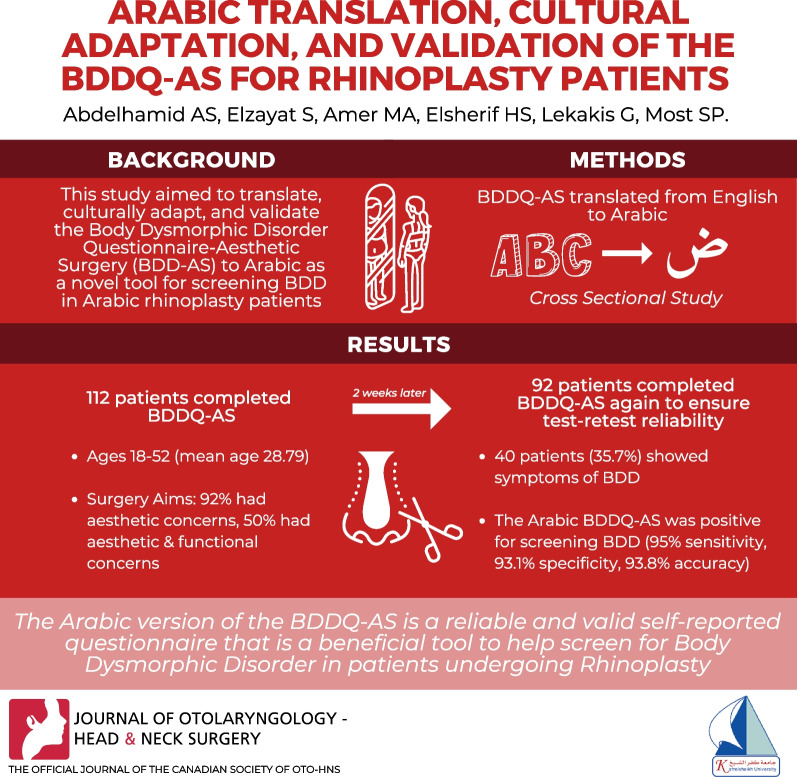

## Introduction

Rhinoplasty occupies an exclusive position in the field of facial plastic surgery. The distinct nasal morphology and central role in the face appear critical for facial harmony and typical psychological issues [1]. Body dysmorphic disorder (BDD) is an obsessive–compulsive disorder (OCD) that affects many people [2]. It is a well-characterized psychological disorder in which individuals have a minor or perceived physical abnormality that results in major impairment in essential functional areas and negatively impacts their quality of life [3]. Studies have shown that these disorders are usually overlooked and underdiagnosed [3, 4].

BDD patients spend much time opposite the mirror, scrutinizing their appearance. It is noteworthy that rhinoplasty is one of the most common cosmetic procedures performed on these patients. However, they are dissatisfied with the outcome of the procedure [3, 5]. The presence of BDD in the rhinoplasty population has been found to be as high as 43% [5], and a recent study at Stanford University found the prevalence to be 32% [6]. Consequently, routine implementation and evaluation of validated BDD questionnaires as screening instruments may optimize patient outcomes [7]. Whereas BDD compromises patients' satisfaction after surgery, rhinoplasty surgeons often overlook this diagnosis preoperatively [8].

The application of a validated BDD screening tool may help identify these patients and prevent adverse outcomes. The Body Dysmorphic Disorder Questionnaire-Aesthetic Surgery (BDDQ-AS) is an existing patient-reported outcome measure, validated for rhinoplasty by Lekakis et al. with sensitivity and specificity of 89.6% and 81.4%, respectively [9]. However, its use among Arabic rhinoplasty patients is nowhere to be applied because of the unavailability of the validated Arabic version. The BDDQ-AS is validated and translated into French, Chinese, Persian, and German [2], 8, 10, 11], but to our knowledge has not been validated and translated to Arabic. Although Arabic is rated fourth, following English, Chinese, and Spanish, with more than 385 million speakers worldwide as the most official languages; besides, it represents one of the United States' six formal languages [12].

Therefore, we intended to see whether the Arabic version of the BDDQ –AS could be translated, culturally adapted, and validated in Arabic-speaking rhinoplasty patients as a screening tool to investigate BDD pervasiveness and intensity in Arabic rhinoplasty individuals.

## Materials and methods

### BDDQ-AS

The BDDQ AS is a short seven-item questionnaire composed of 3 dichotomic questions, "yes/no," and four questions with severity expressed on a five-point Likert scale. The screening is deemed positive if the patients express bother about their appearance and preoccupation due to it (questions 1 and 2 "yes"), as well as a moderately disrupted everyday life (question 7 "yes" or questions 3, 4, 5, or 6 are equal to or greater than "3′′).

### Translation and cultural adaptation

The questionnaire's English into Arabic translation and cultural adaption was conducted with international consensus guidelines to obtain a conceptual translation equivalent to the authentic version [13–15]. We followed standard forward and then back translation of the original BDDQ-AS. Two independent authorized translators created two forwarded translations from English to Arabic. The couple translators were native Arabic speakers who spoke English fluently, translated it into simple Arabic, and then blended. This version was back translated by another translator, oblivious to the content for scrutiny and discrepancy detection of conceptual and cultural content instead of a literal translation.

**Fig. 1:**
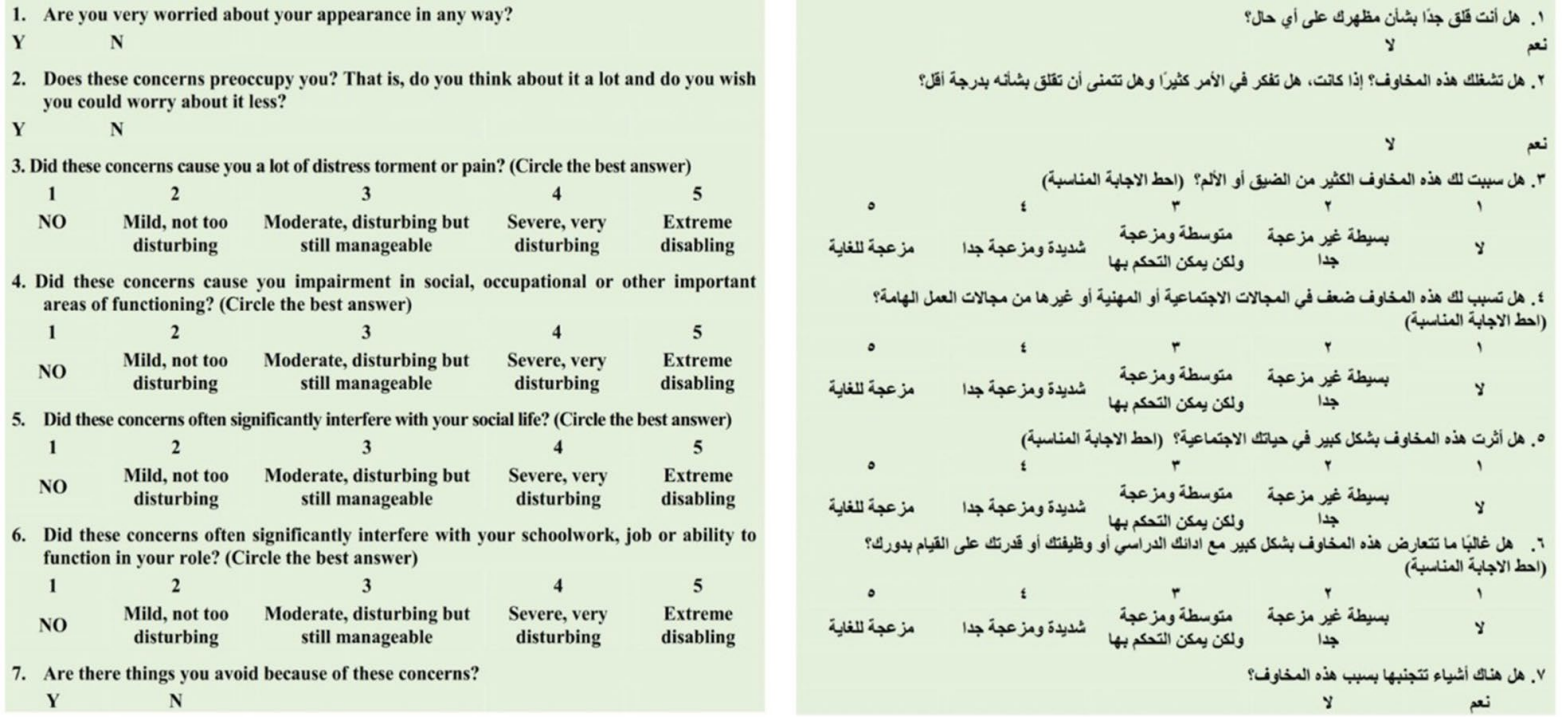
Arabic BDDQ

Before enrolling participants, we conducted pilot testing with two test groups; the first group included ten native Arabic-speaking patients considering rhinoplasty. The patients used a dichotomic scale (clear/unclear) to pinpoint vagueness and for the cognitive debriefing of the created translation. Then, we investigated translation alternatives in terms of a different patient’s interpretation. The second group incorporated three facial plastic surgeons, two psychiatrists, and a dermatologist, who used a Likert scale (four points) to quantify the content validity index (1 = irrelevant to 4 = extremely appropriate). The I-CVI and S-CVI (item and scale content validity indexes) were assessed. Then, the pilot test outcomes were analyzed, along with comments and suggestions regarding the pre-final questionnaire that clarified specifically required adjustments to get the final Arabic version of A-BDD-AS, as illustrated in Fig. 1.

### Study populations

The cross-sectional study was executed at a tertiary referral center, Kafrelsheikh University Hospital, Otorhinolaryngology Department, Egypt, after appropriate institutional review board approval according to the Helsinki Declaration of 1996. One hundred twelve consecutive Arabic seeking rhinoplasty patients were recruited between February and June 2022 to fulfill the proposed Arabic version of the BDD questionnaire at the consult interviews. The participants completed the questionnaire either physically or submitted through an online form. Two weeks later, ninety-two patients were recruited to complete the questionnaire again to ensure test–retest reliability.

We excluded individuals less than 18 years; individuals who did not understand Arabic well or could not interpret the questionnaire were excluded from this study. All were provided written informed consent to be involved in the research. We tested the Arabic version for internal consistency, reliability, validity, sensitivity, and specificity to produce an Arabic validated patient questionnaire.

Cronbach's alpha was disclosed (95% CI) to define internal consistency. The Spearman Correlation Coefficient with P-Value was calculated to study the correlation between the items of A-BDDQ-AS. The discrimination and difficulty variables are defined using item response theory (IRT) analysis. A discriminating variable identifies how sensitive the test is to different levels of symptom severity. The difficulty variable indicates the degree of the perceived nasal issue required to have a 50% chance of selecting a specific score.

### Statistical analysis

The Data were analyzed by IBM SPSS software package version 28. (Armonk, NY: IBM Corp). We used frequency and percentage to describe quantitative data and range (minimum and maximum), mean, and standard deviation to describe qualitative data. The Interclass Correlation Coefficient ICC was used to assess the consistency or reproducibility of quantitative measurements by different observers measuring the same quantity. To measure internal consistency and scale reliability, we could use Cronbach's alpha to check the question's internal consistency. The discrimination and difficulty properties of the scale were evaluated using the item response theory (IRT) model in Stata software (Stata Corp. 2019. Stata Statistical Software: Release 16. College Station, TX: Stata Corp. LLC).

## Results

A total of 112 rhinoplasty patients were recruited for the research. The participants ranged from 18 to 52 years, with a mean age of 28.79. while 61.6% were females. More significant percentages were unmarried (68%), and 73% were non-smokers. 92% of participants had aesthetic concerns concerning surgery aims, with nearly half of patients having functional and aesthetic considerations (Table 1). According to the administered questionnaire, 40 patients (35.7%) showed symptoms of BDD.

**Table 1:** Baseline demographic data of the participants:

	N = 112	%
Gender		
Male	43	38.4
Female	69	61.6
Age (year)		
Mean ± SD	28.79 ± 9.32	
Range	18–52	
Marital status		
Unmarried	68	60.7
Married	35	31.3
Separated	9	8
Smoking		
Yes	30	26.8
No	82	73.2
Aims of surgery		
Functional	8	7.1
Aesthetic	48	42.9
Both	56	50

Content validity index CVI for items 1, 6, and 7 was 1, while that for items 2, 3, 4, and 5 are 0.83 with an overall scale content validity average is 0.83 (acceptable; minimum for all experts is 0.78), and the items were relevant, clear, and straightforward. All experts agreed that the questionnaire is highly relevant in at least three items. The content validity index for Scale S-CVI relevance (if agreement in all scores) is 0.43 (when the score is 3 or 4) is estimated to be 1 (Table 2). Intra Class Correlation ICC of test–retest Reliability ranged from 0.844 to 0.978, which was significant across all scales. ICC's total score was 0.989, indicating excellent short-term internal consistency, reliability, and stability (Table 3).

**Table 2:** I-CVI and S-CVA Arabic version of BDDQ-AS

	ICVI	Number of agreements	Point relevance
Item 1	1	6	1
Item 2	0.83	5	1
Item 3	0.83	5	1
Item 4	0.83	5	1
Item 5	0.83	5	1
Item 6	1	6	1
Item 7	1	6	1
S-CVI/average	0.83	Acceptable	S-CVI relevance = 1
Total agreement	3		
S-CVI/UA	0.43		

I-CVI Content validity index for the item (proportion of experts giving scale relevance of 3 and 4), S-CVI I Content validity index for scale

**Table 3:** Test–retest reliability and internal consistency of items of the Arabic version of BDDQ-AS

Item	Corrected item-total correlation	Cronbach alpha if item removed	ICC (95% CI)	Cronbach alpha (standardized)
Q1	0.791	0.935	0.978 (0.967–0.986)	0.989
Q2	0.753	0.936	0.921 (0.883–0.947)	0.959
Q3	0.912	0.911	0.974 (0.961–0.983)	0.987
Q4	0.926	0.909	0.976 (0.961–0.984)	0.989
Q5	0.94	0.909	0.961 (0.942–0.974)	0.981
Q6	0.9	0.913	0.97 (0.955–0.98)	0.985
Q7	0.789	0.935	0.844 (0.773–0.894)	0.915
Total			0.989 (0.983–0.993)	0.995

Cronbach alpha > 0.7 is acceptable, > 0.8 is good, and > 0.9 is excellent r Correlation coefficient ICC test–retest interclass correlation coefficient CI Confidence interval

According to IRT, the discrimination’s abilities were good (> 2, *p* < 0.05) with acceptable difficulty parameters in all items. (Table 4) This Arabic validation version showed a positive score for screening possible BDD patients with 95% sensitivity and 93.1% specificity with an overall accuracy of 93.8%

**Table 4:** Discrimination and difficulty abilities of A-BDDQ-AS

	Estimate	95% CI	p
Question 1			
Discrimination	3.073	1.11–5.037	0.002*
Difficulty	0.81	0.483–1.137	< 0.001**
Question 2			
Discrimination	4.467	0.8–8.133	0.017*
Difficulty	0.463	0.201–0.725	0.001**
Question 3			
Discrimination	3.999	1.1–6.9	0.007*
Difficulty ≥ 2	0.171	− 0.104–0.446	0.222
Question 4			
Discrimination	4.163	1.092–7.234	0.008*
Difficulty ≥ 2	0.618	0.338–0.898	< 0.001**
Question 5			
Discrimination	8.372	− 4.458–21.2	0.201
Difficulty ≥ 2	0.593	0.343–0.843	< 0.001**
Question 6			
Discrimination	2.961	0.878–5.043	0.005*
Difficulty ≥ 2	1.254	0.813–1.696	< 0.001**
Question 7			
Discrimination	2.336	0.94–3.772	0.001**
Difficulty	1.24	0.775–1.704	< 0.001**

Based on the Hybrid Item Response Theory (IRT)

CI, confidence interval

*p < 0.05 statistical significance; **p ≤ 0.001 high statistical significance

**Fig. 2:**
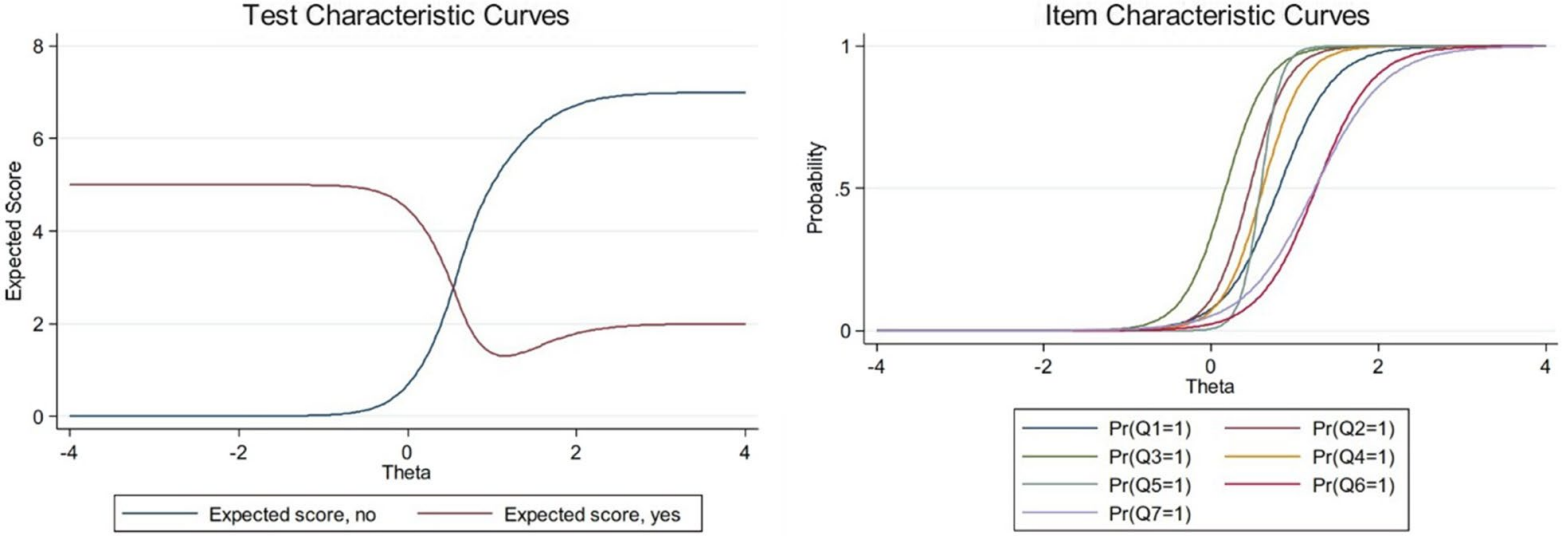
Item and test characteristics curve

Cronbach's alpha coefficient ranged from 0.915 to 0.989 in all items and 0.995 in the total score. Correlation coefficients between items ranged from 0.753 to 0.94. For each deleted item, Cronbach's alpha coefficient was calculated. As the Cronbach's alpha (0.909 to 0.935) remained nearly constant, and none were more significant than the total scale alpha (0.995), it is determined that the tool had high internal consistency.The graphs depicted the characteristics curves for each item and whole test with shifting of the curve toward the more symptoms intensity as illustrated in Fig. 2.

## Discussion

In this study, we successfully translated, adapted, and validated the A- BDDQ-AS as the first study published to date translating the BDD-QS in Arabic. This Arabic form is verified to be equivalent to the original English questionnaire regarding psychometrics and conception. The study found that the questionnaire, as translated, remains a clinically relevant and validated tool for use in Arabic speaking rhinoplasty patients.

Body Dysmorphic Disorder (BDD) is usually under-diagnosed in aesthetic surgery practices. According to studies, 71–76% of those patients seek aesthetic surgery, suggesting that BDD patients are not good candidates for aesthetic surgery [16, 17]. The cosmetic surgery indications should be prudently checked, particularly those with severe symptoms. Nasal aesthetic abnormalities may be linked to negative body image and substantial dissatisfaction with one's body image. In the existing list of psychiatric disorders, body dysmorphic disorder is the only diagnostic section that specifically addresses these issues [18].

In terms of medico-legal arguments that are becoming increasingly important and preventing patients from undergoing unsatisfying surgery, BDD should be recognizable to all facial plastic surgeons [19]. Our work followed the consensus guidelines and strict vigorous steps for cross-cultural adaptation to provide an appropriate version of the BDDQ-AS in Arabic. The translated version from English was straightforward to interpret, according to individuals recruited to be validated, culturally adapted, and adequate analysis of psychometric properties.

The A-BDDQ-AS is a reliable instrument with a high level of internal consistency (Cronbach's alpha 0.995). These outcomes are reasonably comparable to DDDQ-AS in its original English version. The methods utilized in the translation process are a bulwark of content validity. The questionnaire's validity is ensured through psychometric validation, ensuring that the translation does not affect its validity. The significant and positive association among each item of the A-BDDQ-AS is a testimony. The multi-stage validation process was crucial to achieve semantic equivalence of the proposed questionnaire and safeguard that the instrument's authentic content and all concepts were appreciated and adapted to the expectations of the involved persons.

Adopting a validated patient measure in the rhinoplasty field is becoming indispensable as evidence-based medicine spreads [20]. Outcomes in rhinoplasty may be influenced not only by surgical skill, decision-making, and healing but also by the patients' psychological elements, particularly BDD [21].

The ethnic diversity of the population undergoing rhinoplasty is exceptionally high in Arabic-speaking countries. More than 15 countries have Arabic as their native language [12]. In this regard, developing a validated patient-reported outcome measure in Arabic is paramount in rhinoplasty's clinical practice and research purposes. Although validated in a population of aesthetic rhinoplasty patients, its application is not constrained to this population and can be found in various other aesthetic settings [9]. This study reported that 35.8% of rhinoplasty patients expressed BDD symptoms, while there is a lack of publications that describe the prevalence of BDD in Arabic rhinoplasty patients. Further studies discussing the prevalence of BDD in Arabic rhinoplasty candidates are recommended.

The study's sample size is one of its limitations. Nevertheless, on the other hand, many other translation studies have been validated with a comparable or lesser number of participants [22–24]. Furthermore, the psychiatrist consultation for positively screened patients would be the gold standard for confirming BDD diagnosis; this questionnaire has a tremendous role in screening BDD rhinoplasty patients for further referral to the psychiatrist. Another issue, It was only validated in rhinoplasty patients, and the general use of the questionnaire for patients seeking other aesthetic procedures warrants further study.

This version of BDDQ is the first Arabic patients reported outcome measure to detect BDD in candidates for rhinoplasty. It is an easy to interpret, feasible, and less time-consuming questionnaire. It does not take more than three minutes to complete the preoperative screening of BDD, with excellent internal consistency, validity, and reliability.

## Conclusion

This study set out to demonstrate that the Arabic version of the BDDQ-AS is a reliable, valid self-reported questionnaire and is readily used and incorporated in clinical practices. It is a beneficial tool that can help screen Arabic rhinoplasty candidates having body dysmorphic disorder and be utilized in further studies to optimize patient outcomes.

## Data Availability

The datasets used and/or analyzed during the current study are available from the corresponding author on reasonable request.
